# Indicators for Environment Health Risk Assessment in the Jiangsu Province of China

**DOI:** 10.3390/ijerph120911012

**Published:** 2015-09-07

**Authors:** Shujie Zhang, Zhengzheng Wei, Wenliang Liu, Ling Yao, Wenyu Suo, Jingjing Xing, Bingzhao Huang, Di Jin, Jiansheng Wang

**Affiliations:** 1 State Key Laboratory of Resources and Environment Information System, Institute of Geographical Sciences and Resources Research, CAS, Beijing 100101, China; E-Mails: zhangsj@lreis.ac.cn (S.Z.); yaoling@lreis.ac.cn (L.Y.); 2 Policy Research Center for Environment and Economy, Ministry of Environmental Protection, Beijing 100029, China; E-Mails: wei.zhengzheng@prcee.org (Z.W.); Suo.wenyu@prcee.org (W.S.); Xing.Jingjing@prcee.org (J.X.); Huang.bignzhao@prcee.org (B.H.); Jin.di@prcee.org (D.J.); 3 State Key Laboratory of Remote Sensing Science, Institute of Remote Sensing and Digital Earth, CAS, Beijing 100101, China; E-Mail: liuwl@radi.ac.cn

**Keywords:** comprehensive risk of environment and health, pressure-state-response, analytical hierarchy process

## Abstract

According to the framework of “Pressure-State-Response”, this study established an indicator system which can reflect comprehensive risk of environment and health for an area at large scale. This indicator system includes 17 specific indicators covering social and economic development, pollution emission intensity, air pollution exposure, population vulnerability, living standards, medical and public health, culture and education. A corresponding weight was given to each indicator through Analytical Hierarchy Process (AHP) method. Comprehensive risk assessment of the environment and health of 58 counties was conducted in the Jiangsu province, China, and the assessment result was divided into four types according to risk level. Higher-risk counties are all located in the economically developed southern region of Jiangsu province and relatively high-risk counties are located along the Yangtze River and Xuzhou County and its surrounding areas. The spatial distribution of relatively low-risk counties is dispersive, and lower-risk counties mainly located in the middle region where the economy is somewhat weaker in the province. The assessment results provide reasonable and scientific basis for Jiangsu province Government in formulating environment and health policy. Moreover, it also provides a method reference for the comprehensive risk assessment of environment and health within a large area (provinces, regions and countries).

## 1. Introduction

The health risk due to environmental pollution can no longer be ignored, and such environmental pollution has become one of the key risk factors influencing China’s public health. With the improvement of individual living standards, people are paying more attention to the environmental problems affecting human health. In this study, environment refers to the environment (e.g., water, air, soil), the quality of which can be changed by social and economic activities in a short term. This definition excludes some specific natural environment (e.g., air temperature, precipitation and topography). Environment and health risk refers to potential adverse consequences and their severity due to human health risk factors in the environment.

The classical environment and health risk assessment is a four-step method established by the US National Academy of Sciences (NAS) as the norm, mainly including Hazard Identification, Dose-Response Assessment, Exposure Assessment and Risk Characterization [1,2]. According to the pollutant concentration and toxicity and other relevant information, this method generally adds the risks caused through various exposure pathways in a region. This method can describe the hazards of pollutants to human health quantitatively [3,4,5,6,7,8]. It is relatively reliable to evaluate the environment and health risk when the media and pollutants are finite within a small scale area. However, it has some limitations when used in case of multiple media and pollutants within a large scale region. First, the method uses a value (such as average value) to express the concentration of intraregional pollutant usually and this is not scientifically. Second, for multiple pollutants in all media, it is difficult to obtain the data of concentration and toxicity parameters. Third, the direct plus of the health risk of multiple pollutants ignores the interaction effect between pollutants. Fourth, the plus of the health risk of multiple media and pollutants may overestimate the health risk. In addition, the environment and health risk is closely related not only to the environmental quality and exposure pathways but also to the economic development level, investment in environmental protection facilities, government management measures and citizen environmental protection consciousness.

In recent years, many scholars have adopted the indicator system method to evaluate regional sustainable development level, environmental safety, water resources *etc.* Taking the “Pressure-State-Response” (PSR) relationship between human and the environment as the framework, Li *et al.* comprehensively evaluated the environmental quality development trends and change rules in China during 2001 to 2010 by integrating all the environmental monitoring items [9]. On the basis of the “Driving Forces-Pressure-State-Impact-Response” (DPSIR) model, Xiang *et al.* took Guangdong province as an example to conduct a comprehensive assessment on regional water security [10]. Li *et al.* comprehensively assessed the regional harmonious development of environment and health following the human-land relationship [11]. Yao *et al.* used remote sensing and geographical information system (GIS) analysis method to conduct an assessment on ecological and environmental quality of China in 2005, 2008 and 2010 from five aspects: biodiversity, vegetation coverage, water network, land deterioration and environmental pollution [12].

Jiangsu province is one of the developed coastal provinces in Eastern China, and the gross domestic production (GDP) of Jiangsu province was RMB 5.916175 trillion in 2013, accounting for 10.4% of China’s total GDP. With such rapid economic development, a large amount of energy is being consumed, and environmental pollution has become serious. In 2013, the air annual average concentration of PM_2.5_, PM_10_, SO_2_ and NO_2_ in the Jiangsu Province was 73 µg/m^3^, 115 µg/m^3^, 35 µg/m^3^ and 41 µg/m^3^, respectively, and none of the pollutants met the national standards. According to calculations on each day, the days with air quality meeting the standard in the Jiangsu province was only 60.3%, and the number of days of air pollution was 145 in 2013 [13]. This study established multidimensional regional environment and health risk assessment indicator system to qualitatively evaluate the comprehensive environment and health risk of the 58 counties in the Jiangsu province by integrating the data about population, society, economy, enterprise, environment, pollution emission and so on. The assessment result could provide the scientific basis for formulating logical and effective environment health policy by referring the risk differences among counties.

## 2. Methods

### 2.1. Establishment of Comprehensive Risk Assessment Indicators for Environment and Health

Based on the classical Pressure-State-Response (PSR) model [14,15], this study established an indicator system which could reflect comprehensive risk of environment and health. In other words, human social and economic activities generate pollutants which cause pressure on environmental capacity; the pollutants in the environment enter human bodies through various exposure pathways and eventually pose a threat to population health; correspondingly, people respond to health-influencing pollution behavior and take active response measures to protect local environment. The indicator system decomposed an objective problem into three levels: target, criterion and indicator (see Table 1):

1. Target level: indictors which could reflect the comprehensive risk of environment and health in the Jiangsu province.
2. Criterion level: indicators which reflect the comprehensive risk of environment and health in the Jiangsu province from three aspects: Pressure-State-Response.
3. Indicator level: the specific indicators of the criterion level. The pressure index includes eight specific indicators: gross industrial production *per capita*, urbanization level, power consumption per unit of gross industrial production, power consumption per unit area, SO_2_ emission per unit area, NO_2_ emission per unit area, dosage of pesticide per unit area of farmland, and the number of vehicles per unit length of highway. The first two indicators reflect the development degree of the regional economic society, and the next six indicators reflect the pollution emission intensity.

**Table 1:** Indicator system and weighting of comprehensive environment and health risk assessment for Jiangsu province.

Target Level	Criterion Level	Criterion Level Score	Criterion Level Weight	Indicator Level	Indicator Level Score	Indicator Level Weight	Indicator Attribute
Comprehensive risk assessment of environment and health in Jiangsu Province	Pressure index: 0.3864	5	0.3864	Gross industrial production per capita (Yuan/person)	3	0.0412	+
				Power consumption per unit gross industrial production (kwh/Yuan)	3	0.0412	+
				Power consumption per unit area (100 million kwh/km^2^)	3	0.0412	+
				SO_2_ emissions per unit area (tonne/km^2^)	4	0.0549	+
				NO_2_ emissions per unit area (tonne/km^2^)	4	0.0549	+
				Dosage of pesticide per unit area of farmland (kg/hm^2^)	4	0.0549	+
				Number of vehicles per unit length of highway (unit/km)	4	0.0549	+
				Urbanization level (kg/hm^2^)	3	0.0412	+
	State index: 0.3864	5	0.3864	Annual average concentration of NO_2_ (molecules/cm^2^)	5	0.0962	+
				Annual average concentration of SO_2_ (DU/cm^2^)	5	0.0962	+
				Water resources per capita (m^3^/person)	2	0.0385	−
				Total population affected by key enterprises (ten thousand people)	5	0.0962	+
				sensitive population affected by key enterprises (ten thousand people)	3	0.0577	+
	Response index: 0.2308	3	0.2308	GDP per capita (Yuan/person)	2	0.0385	−
				Number of ward beds per ten thousand people (piece/ten thousand people)	3	0.0577	−
				Proportion of individuals in middle schools in resident population	3	0.0577	−
				Household savings deposit per capita (Yuan)	4	0.0769	−

Note: The mark “+”represents the positive indicator, namely, the indicator with a positive effect on the environment and health risk. The mark “−” represents the negative indicator, namely, the indicator with a negative effect on the environment and health risk.

The state index includes five specific indicators: annual average concentration of NO_2_, annual average concentration of SO_2_, water resources *per capita*, total population and total sensitive population affected by key enterprises. The first two indicators reflect the air inhalation exposure, the water resources *per capita* reflect the water exposure, and the last two indicators reflect the population vulnerability. The response index includes four specific indicators. The GDP per capita and household savings deposits *per capita* can reflect local living standards. The higher the living standard is, the stronger the risk response capacity is. The number of ward beds per ten thousand people and the proportion of children in middle school, respectively, reflect the local medical treatment and the culture and education levels; when the medical conditions are better and the educational level is higher, the population’s risk coping capacity will be higher.

### 2.2. Normalization Processing of the Indicator Value

This study took 58 counties in the Jiangsu Province as assessment units. Due to the differences in the numerical measurement unites of the assessment indicators and the large differences between the values of indicators, normalization processing needs to be carried out on each indicator value. Assessment indicators are divided into two types. The first one is the positive indicator, namely, the indicator with the positive effect on environment and health risk. The larger the indicator value is, the higher the environment and health risk is. The normalization calculation formula for the positive indicator is as follows [9]:

(1) $$Q_{i}=\frac{X_{i}-X_{min}}{X_{max}-X_{min}}$$

The second type is the negative indicator, namely, the indicator with the negative effect on environment and health risk; if the indicator value is larger, the environment and health risk will be lower. The normalization calculation formula for the negative indicator is as follows [9]:

(2) $$Q_{i}=\frac{X_{max}-X_{i}}{X_{max}-X_{min}}$$

Where, *X_i_* is the initial value of each indicator; *X_min_* = *min*{*X_i_*,*i* = 1,2,3,…*n*}; *X_max_* = *max*{*X_i_*,*i* = 1,2,3,…*n*}; *n* is the number of assessment units and it is 58 for this study

### 2.3. Determination of Indicator Weight

Analytical Hierarchy Process (AHP) is used to determine the weight coefficient of each indicator. AHP was proposed in the early 1970s by Saaty who was an American operational research expert from the University of Pittsburgh, and AHP is a decision-making analytical method combining qualitative and quantitative analysis [16–18]. Through the consultation with experts in environment and health field, a relative importance value is given to each indicator to determine indicator weight. The indicator importance degree is divided into five levels, with a corresponding score for each one: Absolute importance is five score, four score is very important, three score is relative importance, two score is moderate importance and one score is general importance. According to the indicator scores, the indicator value score matrix is established for the indicators at criterion level and indicator level respectively. The weight of each criterion and each indicator could be obtained on the basis of the consistency check of total sequencing for each level. See Table 1 for the score and weight of each criterion and indicator level.

### 2.4. Comprehensive Risk Assessment of Environment and Health in Jiangsu Province

The comprehensive risk assessment index of environment and health is composed of the pressure index, state index and response index, and the comprehensive assessment index value is the sum of the three indexes. The value of the three indexes is calculated by the sum of the multiplication between each indicator value and corresponding weight. The pressure index is taken as an example and its calculation formula is as follows:

(3) $$V_{p}=\sum_{i=1}^{n}W_{i}Q_{i}$$

Where, *V_p_* is the pressure index, *W_i_* is the weight of No. *i* indicator, *Q_i_* is the value of No. *i* indicator after normalization, and *n* is the number of indicators.

## 3. Data Collection

This study took 58 counties of Jiangsu Province in China as the assessment units and used 17 assessment indicators to describe their state of environment and health risk. Most of the data are from the *Jiangsu Statistical Yearbook 2014* and the *Tabulation on the 2010 Population Census of the People’s Republic of China by County*. The urbanization level indicator was obtained through the ratio between the construction land area and total area in a region. The SO_2_ and NO_2_ concentration data were obtained through the OMI remote sensing monitoring, and the key enterprises data were from the pollution source census. The data statistical analysis was processed using JMP 10.0, and the spatial analysis was processed using ArcGIS 10.0 and ENVI 4.6.

### 3.1. Statistics of Total Population Affected by Key Enterprises

Enterprise presents potential health risk to the population distributed in its vicinity. If there are more enterprises in a region and the population in the surrounding area is greater, the environment and health risk in this region will be higher. This study collected 4751 key enterprises from 12 industries generally monitored by China. The 58 counties in the Jiangsu province were used as the spatial statistical units. The total population and total sensitive population affected by key enterprise were counted according to enterprise health protection distance standards. If there is not health protection distance standard, the total population and total sensitive population were counted within the scope of 1000 m around an enterprise.

The population data is a kind of statistical data. In order to ensure the accuracy of the affected population, spatial distribution processing of population data was conducted. First, we linked the statistical data in the table form and the administrative data with geographic coordinates. Thus, the population spatial distribution data was established. The statistics for the total population affected by key enterprise were computed by referencing land use data. The land use data is from the Data Sharing Infrastructure of Earth System Science (http://www.geodata.cn/Portal/index.jsp). This study combined the land use data into seven types: forest, grassland, farmland, water body, desert, town and village. Because the population should be only distributed in towns and villages, the calculation formula of the total population affected by key enterprise is as follows:

(4) $$Pop_{affected}=\frac{TPop_{total}}{TArea_{total}}\text{*}\sum_{n=1}^{n}TArea_{affected}+\frac{CPop_{total}}{CArea_{total}}\text{*}\sum_{n=1}^{n}CArea_{affected}$$

where *Pop_affected_* is the total population affected by key enterprises for a county, *TPop_total_* is the total urban population of a county, *TArea_total_* is the total urban area of a count, *CPop_total_* is the total rural population of a county, *CArea_total_* is the total rural area of a county, *TArea_affected_* is the urban area affected by key enterprises for a county, *CArea_affected_* is the rural area affected by key enterprises for a county, and *n* is the number of enterprises. However, the total urban and rural sensitive population (children less than or equal to 14 years old and the elderly population greater than or equal to 65 years old) of a county could not be obtained. Therefore, the calculation formula of the total sensitive population affected by key enterprise is as follows:

(5) $$SPop_{affected}=\frac{SPop_{total}}{Area_{total}}\text{*}\sum_{n=1}^{n}Area_{affected}$$

where *SPop_affected_* is the total sensitive population affected by the key enterprises for a county, *SPop_total_* is the total sensitive population of a county, *Area_total_* is the total area of urban and rural for a county, and *Area_affected_* is the sum of urban and rural area affected by key enterprises for a county.

**Figure 1:**
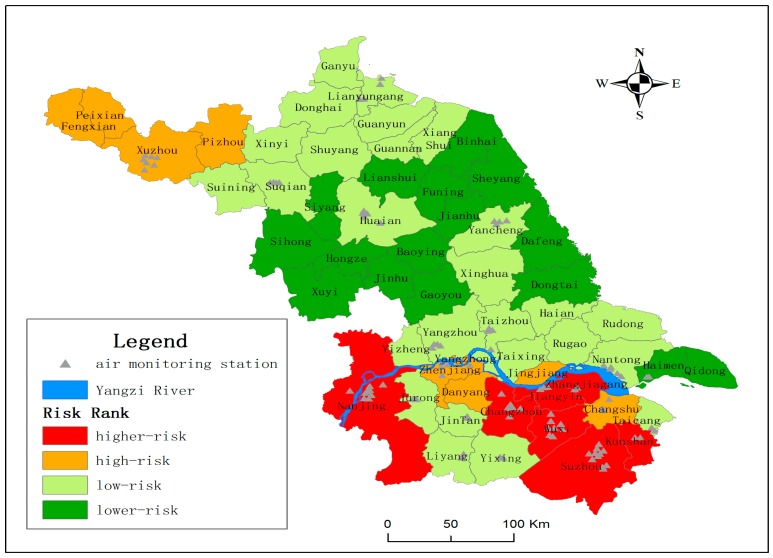
Comprehensive risk distribution of environment and health in the Jiangsu province.

### 3.2. SO_2_ and NO_2_ Concentration Data Acquisition

For the 58 counties in the Jiangsu province, only 23 counties have air monitoring stations. As Figure 1 shows, the spatial distribution of the air monitoring stations is very uneven. Air monitoring stations are sparse in central and northern Jiangsu Province. For the counties with no air monitoring stations, if the SO_2_ and NO_2_ data are predicted by applying spatial interpolation methods, the prediction results would be unreliable due to the limited monitoring stations. This study used the OMI Total Columns Nitrogen Dioxide and Total Column Sulphur Dioxide products (http://disc.sci.gsfc.nasa.gov/Aura/data-holdings/OMI). The level of the data product is 3, and the product name for NO_2_ is OMNO2d, and the name is OMSO2e for SO_2_. The sensor of zone monitoring instrument (OMI) is on the Aura satellite which was launched by NASA (National Aeronautics and Space Administration) on 15 July 2014. OMI was jointly manufactured by Netherlands Institute for Air and Space Development (NIVR) and Finnish Meteorological Institute (FMI), which is used to detect the atmospheric composition, for example O_3_, NO_2_, SO_2_, HCHO, BrO, OCIO, AOD, *etc.* The spatial resolution for SO_2_ and NO_2_ is 0.25° × 0.25° (Lat/Lon).

## 4. Results

Based on the 17 environment and health risk assessment indicators, the relevant data for the social economy, pollution emission intensity, environmental quality state, living standards, medical health and cultural education were used to compute the comprehensive risk index of environment and health of the 58 counties in the Jiangsu province. The comprehensive environment and health risk index was divided into four levels (see Table 2 for the level division criterion) according to the equipartition method; Level 1 denotes higher-risk areas; Level 2 is relatively high-risk areas; Level 3 is relatively low-risk areas and Level 4 is lower-risk areas. There are more counties at Levels 3 and 4. Therefore, Table 3 only shows the risk indexes of counties at Level 1 and 2 and their ranking position among the 58 counties. The risk level spatial distribution of the 58 counties in Jiangsu province is shown in Figure 1. In general, higher-risk areas are located in the economically developed southern region of Jiangsu province.

**Table 2:** The division criterion for comprehensive risk of environment and health.

Risk Level	Comprehensive Risk Index	Description
Level 1: higher-risk areas	50–60 scores	The environment and health risk is highest. Although the risk response capacity is strong, the pollution pressure is great and the environmental state is very poor.
Level 2: high-risk areas	40–50 scores	The environment and health risk is higher, the environmental state is poor, and the pollution pressure and risk response capacity have different levels.
Level 3: low-risk areas	30–40 scores	The environment and health risk is lower, and the pollution pressure, environmental state and risk response capacity are all scored as general. Therefore, these areas have harmonious development relationship between environmental and population health.
Level 4: lower-risk areas	20–30 scores	The environment and health risk is the lowest, the pollution pressure is small, the environmental state is good, and the risk response capacity is corresponding weakest.

The higher-risk area includes seven counties: Jiangyin, Wuxi, Suzhou, Changzhou, Kunshan, Zhangjiagang and Nanjing. All of these seven counties are all located in the economically developed southern region of Jiangsu province. These counties have highly concentrated urban population and are highly industrialized. Therefore, the pressure index and state index are all very high. Except for the state index of Kunshan, which ranks No.16, the pressure indexes and state indexes of the other counties all rank in top 10 in the province. However, in these counties, the economy is developed, the government’s investment in medical health and education is high. At the same time, the household savings per capita and the living standard is high. Therefore, the overall risk response capacity is strong. With the exception of Kunshan city, all the response indexes rank in top 10. Compared with other counties, Kunshan has a slightly higher state index, slightly poor risk response capacity and higher comprehensive environment and health risk. So, we should pay more attention to environment and health problems of Kunshan. In general, for improving the environment and health risk, environment and health risk prevention of these counties need to start from the source of the pollution by optimizing the spatial layout of industrial, reducing internal pollution sources and *etc.*

**Table 3:** Counties with environment and health comprehensive risk at level 1 and 2.

County	Pressure Index	Pressure Ranking	State Index	State Ranking	Response Index	Response Ranking	Comprehensive Risk Index	Comprehensive Risk Level
Jiangyin	25.91	2	23.77	4	9.07	3	58.75	1
Wuxi	18.30	7	31.31	1	8.14	2	57.74	1
Suzhou	18.67	5	28.35	3	9.90	6	56.91	1
Changzhou	13.33	10	30.58	2	10.08	9	53.99	1
Kunshan	22.96	3	17.87	16	11.98	14	52.81	1
Zhangjiagang	25.97	1	21.09	8	4.89	1	51.94	1
Nanjing	18.39	6	23.41	5	10.02	7	51.82	1
Changshu	19.04	4	17.28	17	9.29	4	45.62	2
Peixian	6.27	28	20.96	9	18.05	55	45.28	2
Xuzhou	8.42	14	22.45	7	13.88	24	44.75	2
Danyang	7.40	20	22.66	6	14.08	25	44.13	2
Jingjiang	13.89	9	17.92	15	10.04	8	41.85	2
Fengxian	5.91	32	19.19	12	16.49	44	41.59	2
Zhenjiang	9.57	12	19.12	13	12.31	18	41.00	2
Pizhou	5.40	38	17.00	18	17.72	49	40.11	2

The high-risk area includes eight counties of Jiangsu province: Three counties (Changshu, Danyang and Zhenjiang) are located in the economically developed southern region; one county (Jingjiang) is located in the central region with moderate economic strength; four counties (Peixian, Xuzhou, Fengxian and Pizhou) are located in the northern region with relatively weak economic strength. These eight areas also have better regional advantages, rapid economic development, large population quantities, a large number of enterprises with high energy consumption and pollution. Therefore, the state indexes of these counties are all high and rank in top 20 in the Province. Changshu, Jingjiang and Zhenjiang have higher pressure indexes (ranking No.4, No.9 and No.12, respectively) and stronger risk response capacities (ranking No.4, No.8 and No.18, respectively); Xuzhou and Jingjiang have moderate pressure (ranking No.4, No.20, respectively) and risk response capacities (ranking No.24, No.25, respectively); Peixian, Fengxian and Pizhou have less pressure (ranking No.28, No.32 and No.38, respectively) and weaker risk response capacities (ranking No.55, No.44 and No.49, respectively).

The low-risk area includes 27 counties with relatively dispersive spatial distribution (see Figure 1). At low-risk level, the counties with higher pressure index include Yangzhou, Taicang, Yixing, Jintan, Xiangshui and Hai’an (all ranking among top 20 in Jiangsu Province). The counties which have higher state index and rank in top 20 in the Province include Yangzhou, Ganyu, Taizhou and Yangzhong. The other counties have harmonious relationship between environmental development and population health.

The lower-risk area includes 16 counties which located in the north—central region of Jiangsu province. All of these lower-risk counties have very low pressure indexes and state indexes, except for Haimen with higher pressure index (ranking No.15 in the province). All the risk response capacity indexes of these 16 counties are also corresponding low. In a word, these counties have good environment conditions and low health risk.

**Figure 2:**
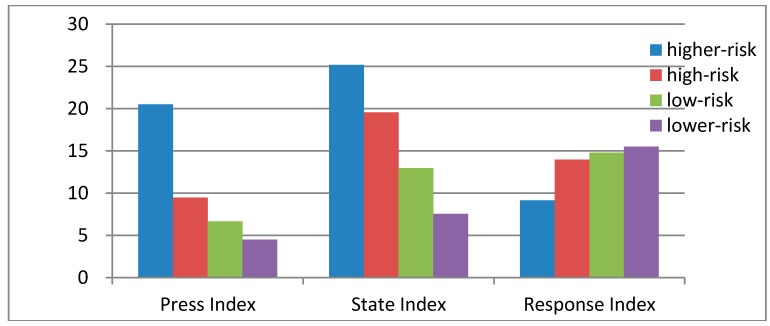
Average risk indexes for four type counties.

According to average value of all sub-indexes (pressure index, state index, response index) in Figure 2, the pressure index of higher-risk area is obviously higher than that of the area at the other three levels, which indicates that the economic development level, urbanization level and pollution emission intensity in higher-risk area are obviously higher than those of the areas at the other three levels. The high environment pressure directly leads to the high risk state. In the counties with higher-risk, because of the strong financial strength, the government’s investment in the cultural education and public health is high. So the risk response capacity is corresponding stronger than that of the areas at the other three levels.

## 5. Discussion and Conclusions

The classical four-step environment and health risk assessment method quantitatively evaluates the potential risk of pollutants to human health by plus the risks caused by pollutants through various exposure pathways. The pollutant concentration, toxicity, exposure parameters and other relevant data must be known. This method is more suitable for evaluating health risk within an area at small scale, especially suitable for field. However, it has some limitation when evaluating environment and health risk at large scale region. Adopting the indicator system method to comprehensively evaluate the environment and health risk for large area (provinces, regions and countries) is still at exploratory stage. On the basis of the Pressure-State-Response framework, this study established an indicator system which can reflect the comprehensive environment and health risk of a region. The indicator system covers relevant indicators which include social and economic development, pollution emission intensity, air inhalation pollution exposure, water exposure, population vulnerability, living standards, medical and public health, cultural education and so on. A weight coefficient is given to each indicator through the analytic hierarchy process, and then the assessment of the comprehensive environment and health risk of 58 counties in the Jiangsu province was conducted according to the statistical and remote sensing data. The 58 counties were divided into four types. The higher-risk and high-risk areas are mainly located in the economically developed region along the Yangtze River. The distribution of low-risk areas is relatively dispersive, and lower-risk areas are mostly located in the middle region with relatively weak economy in the province. This result showed that, for most of the counties in the Jiangsu province, the more developed the counties are, the higher the environment and health risk is. In future development of Jiangsu, while maintaining economic growth, all counties should pay attention to the adjustment of industrial structures, and the environmental protection consciousness should be strengthened. For the counties with high pressure index, pollutant emissions should be reduced largely. For the counties with high state index, the environment management needs to be strengthened in time. Meanwhile, in order to improve the risk response capacity, government’s investment in environmental protection facilities, medical health, cultural education and other aspects should be increased. The comprehensive risk of environment and health would be reduced by taking the above measures. On the other hand, we can take active measures to monitor the environment before the outbreak of health events.

Due to the limited data availability, the environment and health comprehensive risk assessment indicator system proposed in this study has some limitations. In future work, if we could collect more pollutant emission data, we would expand the indicator system to improve the reliability of the assessment results. For instance, the drinking water quality state, heavy metal content in the soil and other exposure parameters are very important indicators to reflect the comprehensive environment and health risk, however, these indicators had to be omitted because the data are very difficult to obtain. In addition, the indicators selected in this study do not include morbidity, death rates, mainly because that the health risk evaluated in this study is potential risk caused by environmental pollution, but not the health outcome.
